# COVID-19 Co-infection with *Legionella pneumophila* in 2 Tertiary-Care Hospitals, Germany

**DOI:** 10.3201/eid2705.203388

**Published:** 2021-05

**Authors:** Hedda L. Verhasselt, Jan Buer, Jutta Dedy, Renate Ziegler, Joerg Steinmann, Frank Herbstreit, Thorsten Brenner, Peter-Michael Rath

**Affiliations:** University of Duisburg-Essen, Essen, Germany (H.L. Verhasselt, J. Buer, J. Dedy, F. Herbstreit, T. Brenner, P.-M. Rath);; Paracelsus Medical University, Nuremberg, Germany (R. Ziegler, J. Steinmann)

**Keywords:** SARS-CoV-2, COVID-19, coronavirus, 2019 novel coronavirus disease, severe acute respiratory syndrome coronavirus 2, zoonoses, coronavirus disease, viruses, coronavirus infection, pandemics, pneumonia, Legionella pneumophila, co-infection, Germany

## Abstract

We describe screening results for detection of co-infections with *Legionella pneumophila* in patients infected with severe acute respiratory syndrome coronavirus 2. In total, 93 patients were tested; 1 was positive (1.1%) for *L. pneumophila* serogroup 1. Co-infections with *L. pneumophila* occur in coronavirus disease patients and should not be missed.

Severe acute respiratory syndrome coronavirus 2 (SARS-CoV-2) infection, which causes coronavirus disease (COVID-19), is characterized by severe respiratory distress, fever, and cough. High death rates, especially in older persons and those with underlying health conditions, have been described (*1*). According to World Health Organization guidelines and public health agencies, persons with cardiovascular disease, chronic respiratory disease, diabetes, and cancer are considered to be at increased risk for severe COVID-19. Moreover, the risk of becoming severely ill increases with age >60 years (https://www.who.int/publications/m/item/covid-19-and-ncds).

Groups at risk are largely the same for COVID-19 and Legionnaires’ disease (LD), a severe and potentially fatal pneumonia caused by *Legionella* spp. These bacteria are found in many environments, including complex building water systems. In Europe and North America, *Legionella* spp. account for ≈1%–16% of all community-acquired pneumonias that require hospitalization (*2*); in 2017, the overall notification rate was 1.8/100,000 population for the European Union/European Economic Area (European Centre for Disease Prevention and Control, https://www.ecdc.europa.eu/en/publications-data/legionnaires-disease-annual-epidemiological-report-2017). *L. pneumophila* is responsible for >90% of LD cases; specifically, serogroup 1 causes 70%–80% of LD cases in the United States and Europe (*3*). Currently, the Centers for Disease Control and Prevention and the European Society of Clinical Microbiology and Infectious Diseases Study Group for Legionella Infections give warning of increased risk for *Legionella* spp. infections resulting from stagnant or standing water in plumbing systems after the temporary shutdown of buildings and reductions in normal water use (*4*,*5*). A single person with SARS-CoV-2 revealed *L. pneumophila* co-infection in the context of travel (*6*). This case underlines the importance of making differential diagnoses during the COVID-19 pandemic by diagnostic microbiology to identify other infectious microorganisms causing similar symptoms.

In this retrospective analysis, we evaluated the co-occurrence of infections with *L. pneumophila* in patients infected with SARS-CoV-2. We performed urine antigen tests for detection of *L. pneumophila* serogroup 1 (BinaxNOW Legionella; Abbott Rapid Diagnostics Germany GmbH, https://www.de.abbott). We analyzed urine samples from 93 patients from 2 tertiary-care hospitals in Germany: University Hospital Essen, Essen, and General Hospital Nürnberg, Nuremberg. This retrospective study was approved by the Ethics Committee of the Medical Faculty at the University of Duisburg-Essen, Germany (approval no. 20–9335-BO).

The cohort was mostly male (71.0%) and had a mean age of 65 years; 90% had symptoms of pneumonia (Table). All were hospitalized, and 38.7% received mechanical ventilation. More than one third of the cohort had >2 underlying conditions and reflected the groups at risk for infection with *Legionella* spp.

**Table:** Demographics and underlying conditions of patients with COVID-19 examined for *Legionella pneumophila* urine antigen, Germany

Characteristic	Value
Total	93 (100.0)
Negative for *L. pneumophila* serogroup 1 antigen	92 (98.9)
Positive for *L. pneumophila* serogroup 1 antigen	1 (1.1)
Average time between admission and *Legionella* antigen test processing	2.6 d (mean), 1 d (median)
Legionella-specific culture† performed/positive	18 (19.4)/0
Legionella nonspecific culture performed/positive	35 (37.6)/11 (31.4)
Multiplex PCR‡ performed/positive	31 (33.3)/5 (16.1)
Clinical symptoms typical for COVID-19§	60 (90.0)
Hospitalized	93 (100)
Transferred from other hospital	35 (37.6)
Treated in intensive care unit	40 (43.0)
Mean age, years	65
Sex	
M	66 (71.0)
F	27 (29.0)
Invasive mechanical ventilation	36 (38.7)
Extracorporeal membrane oxygenation	17 (18.3)
Mortality	30 (32.3)
Underlying conditions	
Cardiovascular disease	50 (53.8)
Diabetes	28 (30.1)
Chronic respiratory disease	13 (14.0)
Cancer	10 (11.0)
Other: rheumatism, Parkinson's disease	17 (18.3)
Addictions: alcohol, nicotine	7 (7.5)
Solid organ transplantation: lung	1 (1.1)
None	15 (16.1)
1 underlying condition	55 (59.1)
2 underlying conditions	29 (31.2)
>2 underlying conditions	7 (7.5)

*Values are no. (%) except as indicated. †Legionella BMPA selective agar (Thermo Scientific, https://www.thermofisher.com).  ‡Unyvero P50 pneumonia application (Curetis GmbH, https://curetis.com) or ampliCube Respiratory Panel 1 (Mikrogen Diagnostic, https://www.mikrogen.de). §Data available for 67 patients.

We detected 1 *L. pneumophila* serogroup 1 antigen in the entire cohort (1.1%). The patient with *L. pneumophila* serogroup 1 co-infection was a 41-year-old man with severe acute respiratory deficiency syndrome and bronchial asthma as underlying disease; he initially came to the hospital with fever, cough, and dyspnea and had no recent travel history. Before admission to the University Hospital, he was treated with azithromycin and ceftriaxone for 4 days, until a switch to levofloxacin on day 1 after first diagnosis of LD in the referral hospital. In the University Hospital, urine antigen test was still positive, and detection of *Legionella* spp. DNA from bronchoalveolar fluid revealed a PCR cycle threshold value of 34 (ampliCube Respiratory Panel 1; Mikrogen Diagnostic, https://www.mikrogen.de), which was assessed as negative. To exclude a false-positive antigen test result, we retested this specific urine sample after boiling for 5 min and centrifugation (5 min at 12,000 × *g*), which yielded a positive result again (*7*). As of July 2020, the patient was still critically ill, receiving mechanical ventilation and intravenous levofloxacin (500 mg 2×/d; day 6 of levofloxacin treatment).

Xing et al. reported *L. pneumophila*, detected by indirect immunofluorescence in 20% of COVID-19 patients, as the second most prevalent bacterium causing respiratory disease (Q. Xing et al., unpub. data, https://www.medrxiv.org/content/10.1101/2020.02.29.20027698v2). However, cross-reactivity of indirect immunofluorescence tests with other bacterial species has been described. Antibody titers without follow-up should be interpreted with caution because antibodies can be generated even after mild infections and can persist over years.

In view of epidemiologic data, detection of only *L. pneumophila* serogroup 1 antigen in urine is a suitable diagnostic approach for outpatient-acquired and travel-associated pneumonia, with varying sensitivity and specificity (*8*). The false-negative rate of this diagnostic approach is low because antigen excretion starts 24 hours after first symptoms and generally persists for weeks, and in rare cases even months (*9*); positive urine antigen tests can be found after initiation of antimicrobial drug treatment. However, pretest probability of *L. pneumophila* pneumonia should be reasonably high to have clinical utility (*10*).

The findings from our small cohort study in 2 geographically distinct areas in Germany indicate that co-infections with *L. pneumophila* serogroup 1 can occur in patients with COVID-19. Clinicians treating patients positive for SARS-CoV-2 should be aware of possible co-infections with *L. pneumophila* and should use appropriate diagnostic approaches.
